# Bronchial Asthma and COVID-19: Etiology, Pathological Triggers, and Therapeutic Considerations

**DOI:** 10.3390/pathophysiology31020020

**Published:** 2024-05-27

**Authors:** Anna Starshinova, Anastasia Borozinets, Anastasia Kulpina, Vitaliy Sereda, Artem Rubinstein, Igor Kudryavtsev, Dmitry Kudlay

**Affiliations:** 1 Almazov National Medical Research Centre, 197341 St. Petersburg, Russia; igorek1981@yandex.ru; 2 Medical Department, I.M. Sechenov First Moscow State Medical University, 197022 Moscow, Russia; 3 Medical Department, Saint Petersburg State Pediatric Medical University, 194100 St. Petersburg, Russia; asya.starshinova@mail.ru; 4 Medical Department, Saint Petersburg State University, 199034 St. Petersburg, Russia; seredavitaly@bk.ru; 5 Department of immunology, Institution of Experimental Medicine, 197376 St. Petersburg, Russia; arrubin6@mail.ru; 6 Institute of Immunology FMBA of Russia, 115478 Moscow, Russia; d624254@gmail.com; 7 Department of Pharmacognosy and Industrial Pharmacy, Faculty of Fundamental Medicine, Lomonosov Moscow State University, 119991 Moscow, Russia

**Keywords:** bronchial asthma, COVID-19, SARS-CoV-2, predisposing factors, immune response, targeted therapy

## Abstract

Bronchial asthma (BA) continues to be a difficult disease to diagnose. Various factors have been described in the development of BA, but to date, there is no clear evidence for the etiology of this chronic disease. The emergence of COVID-19 has contributed to the pandemic course of asthma and immunologic features. However, there are no unambiguous data on asthma on the background and after COVID-19. There is correlation between various trigger factors that provoke the development of bronchial asthma. It is now obvious that the SARS-CoV-2 virus is one of the provoking factors. COVID-19 has affected the course of asthma. Currently, there is no clear understanding of whether asthma progresses during or after COVID-19 infection. According to the results of some studies, a significant difference was identified between the development of asthma in people after COVID-19. Mild asthma and moderate asthma do not increase the severity of COVID-19 infection. Nevertheless, oral steroid treatment and hospitalization for severe BA were associated with higher COVID-19 severity. The influence of SARS-CoV-2 infection is one of the protective factors. It causes the development of severe bronchial asthma. The accumulated experience with omalizumab in patients with severe asthma during COVID-19, who received omalizumab during the pandemic, has strongly suggested that continued treatment with omalizumab is safe and may help prevent the severe course of COVID-19. Targeted therapy for asthma with the use of omalizumab may also help to reduce severe asthma associated with COVID-19. However, further studies are needed to prove the effect of omalizumab. Data analysis should persist, based on the results of the course of asthma after COVID-19 with varying degrees of severity.

## 1. Introduction

Today, the problem of bronchial asthma (BA), as well as other chronic diseases, is of particular importance due to the emergence of a new coronavirus infection (COVID-19) in March 2020 [1,2].

The World Health Organization has recently reported the number of asthma patients in the world to be more than 339 million. Experts predict that the number of asthma patients will increase to 400 million by 2025 [3].

The disease is associated with a high quality of life, but mortality is associated with poor quality of life and health care, according to research from 2019. Lack of control or partial control of asthma symptoms, even when it is mild, are serious risk factors for exacerbations and disease severity, especially during different seasons in COVID-19 incidence [4,5].

Developed countries which may be associated with increased urbanization and a high standard of living demonstrate a high prevalence of asthma. According to studies, the countries with the highest prevalence of asthma are Australia (21.5%), Sweden (20.2%) the United Kingdom (18.2%), the Netherlands (15.3%), and Brazil (13.0%); however, the United States and Canada were excluded. BA is diagnosed significantly less frequently in Vietnam (1.0%), Bosnia and Herzegovina (1.4%), and China (1.4%) [5].

It is known that asthma is associated with poor quality of life, significant social burden, and risk of adverse outcomes, including disability and death [6,7,8]. In the United States, African Americans, Puerto Ricans, and Cubans have the highest mortality rates [5].

It should be taken into account that even a mild course of asthma can be characterized by severe exacerbations. Triggers that induce airway inflammation or acute bronchospasm lead to an exacerbation of BA, where viral respiratory infections are of particular importance. The most significant among them are allergens, meteorological factors, physical activity, and intake of some medications [6]. A special role in the genesis of BA exacerbations is attributed to respiratory viral infections [7].

At the same time, the influence of SARS-CoV-2 virus as a trigger can provoke the appearance of asthma symptoms, worsening of asthma course, and appearance of additional symptoms under a COVID-19 mask [1].

The severity low control of clinical symptoms and unfavorable outcomes of asthma are usually associated with non-compliance of treatment with scientifically based domestic and international recommendations [9,10]. Meanwhile, the impact of a novel coronavirus infection (COVID-19) on the course of asthma during the exacerbation of infection and after reconvalescence is not fully understood. The available data on this issue do not fully describe this problem and are often contradictory in nature [11]. The aim of the review was to determine the peculiarities of the course and immune response in patients with bronchial asthma before and after COVID-19.

## 2. Etiology and Pathogenesis of Asthma

To date, the diagnosis of asthma is a complex and multistep process that requires not only a careful collection of anamnesis, but identification of all possible risk factors, including genetic predisposition, contributing to the development and severe course of asthma [12,13]. With the emergence of COVID-19, we have a new trigger factor that affects not only the activation of asthma but also determines the severity of the course of both COVID-19 itself and the severity of asthma [12,14].

Compliance with all the principles of diagnosis and differential diagnosis of asthma is more likely to ensure the prescription of correct and adequate therapy, guaranteeing not only the absence of exacerbations and long-term remission but also the quality of life of patients without the need for inhalation therapy [15].

**Figure 1:**
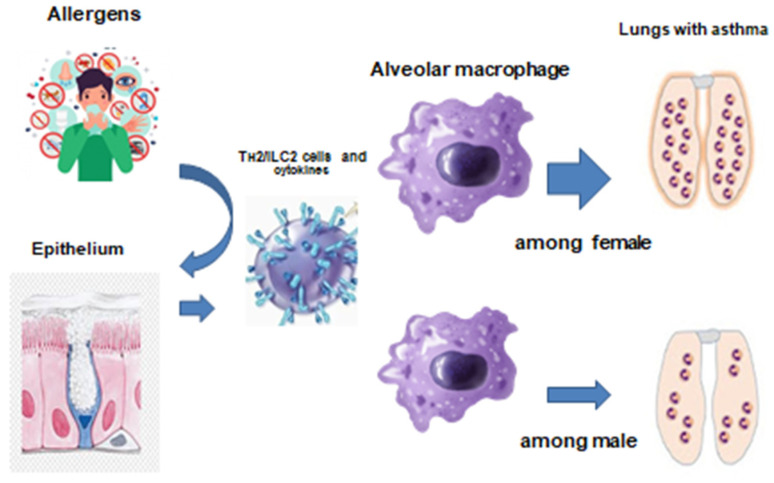
The etiology and pathogenesis of bronchial asthma [17].

It is now known that asthma is characterized by the chronic development of inflammation, proceeding with the participation of mast cells, eosinophils, and T-lymphocytes (Figure 1), and the release of large amounts of inflammatory mediators. Inflammation of the airways causes their hyperreactivity, bronchial obstruction, and respiratory symptoms [15,16].

According to the literature, there is a gene-mediated interaction where a susceptible host is exposed to environmental factors. Allergens are capable of generating IgE and sensitization occurs. However, exactly why the airways of some individuals are susceptible to allergic factors has not been established [16].

Angiotensin converting enzyme 2 (ACE2) and transmembrane serine protease 2, which are receptors on airway epithelial cells, have been identified by single-cell RNA sequencing or immunostaining. It was found that the expression levels of these molecules varied, depending on the type, function, and location of airway epithelial cells. Currently, we know that the intestinal epithelial cells, secretory cells, all factory epithelial cells, and alveolar epithelial cells vary according to different factors (depending on age, sex, or comorbidities) [18].

The SARS-CoV-2 virus has also been determined to cause widespread organ damage, associated with ACE2 expression. The SARS-CoV-2 virus enters cells, expressing ACE2 or cells, expressing other coronavirus receptors. It may be aminopeptidase N and dipeptidyl peptidase 4 (DPP4). This fact suggests that ACE2 is a cellular receptor for SARS-CoV-2 [19]. Ex vivo experimental studies have shown that the respiratory epithelial cells, infected by SARS-CoV-2, produce chemokines and cytokines, and attract inflammatory cells to target organs. This IFN signaling pathway is a critical pathway for the body’s defense when other viral infections. Currently, research into the pathophysiological mechanisms of the influence of SARS-CoV-2 are ongoing [18].

Also, it can be hypothesized that the key role in the development of bronchial asthma, especially after a COVID-19 infection, and the key role of the immune response belong to the background of a genetic predisposition to the development of bronchial asthma. New research findings add to our knowledge of the mechanisms that are involved in abnormal immune system functioning in the development of asthma after COVID-19.

**Figure 2:**
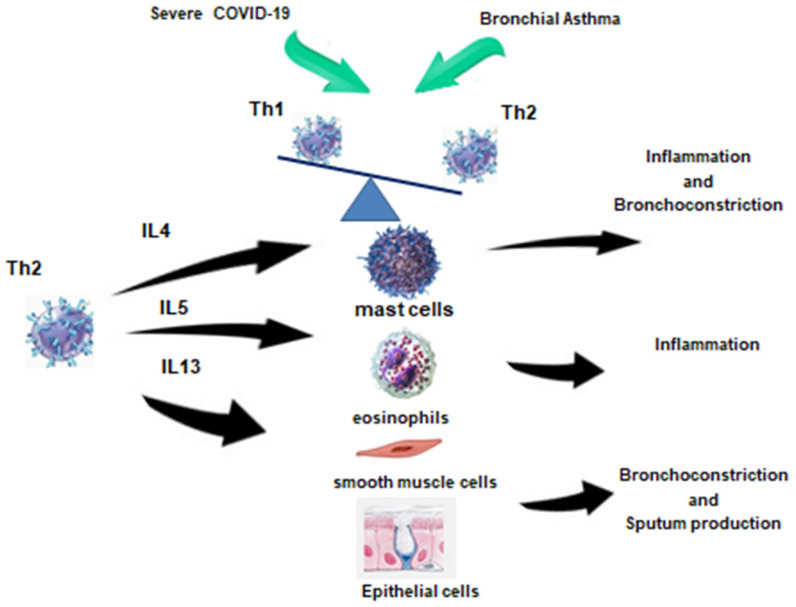
Immune response in patients with COVID-19 and bronchial asthma [17].

One of the debated issues remains the possible influence of Th2 inflammation on the risk of severe COVID-19 (Figure 2).

In the peripheral blood of patients with Th2 endotype of BA, there is an increase in blood Th2 with a simultaneous decrease in Th1, which is also observed in severe COVID-19. In the case of SARS-CoV-2, this phenomenon may be compensatory in order to suppress hyperinflammation, whereas in asthma, Th2 cells contribute to allergic reactions. Thus, in the period of reconsolidation after severe COVID-19, the development of Th2 BA may be provoked. Th2 in this case will contribute to the development of inflammation, bronchospasm, and sputum hyperproduction, acting on the corresponding effector cells depicted in the figure.

## 3. Immunologic Features of Bronchial Asthma Development

Bronchial inflammation plays a key role in the pathogenesis of asthma. The pattern of inflammation, that forms in the bronchial wall of the airways, does not always correlate with the severity or duration of the bronchial asthma [5,6].

Recently, a link between innate and adaptive immunity and the interactions of their cells has attracted significant interest in pathophysiology of this disease [18,19]. Airway inflammation in asthma may represent a loss of normal balance between two “opposing” Th-lymphocyte populations.

Th1 and Th2 have different cytokine profiles. Th1 cells produce IL-2 and interferon-γ (IFN-γ) which play a crucial role in cellular defense mechanisms in response to infection, although Th2 has the ability to secrete the cytokines (IL-4, -5, -6, -9, and -13) that may mediate allergic inflammation [20].

The modern “hygiene hypothesis” of asthma demonstrates how this cytokine imbalance may explain the increase in asthma prevalence in Western countries. This hypothesis is based on the assumption that the neonatal immune system tends to produce Th2 cytokines. After birth, various infections can activate Th1 responses, leading to changes in the Th1/Th2 ratio causing a corresponding balance [21,22].

Evidence suggests that the incidence of asthma is reduced by certain infections (tuberculosis, measles, or hepatitis A), exposure to other children (e.g., having older siblings and early kindergarten enrollment), and increases with early antibiotic use [18,23].

Moreover, the absence of the following events may lead to a predisposition to Th2-associated allergic reactions. For example, cytokines secreted by Th2 can induce the production of IgE to various environmental antigens such as house dust mites, cockroaches, and possibly cat hair [24,25,26]. 

Eosinophils are thought to play an important role in the pathogenesis of asthma through secretion of inflammatory mediators [27,28]. In severe cases of bronchial asthma, the development of eosinophils play a major role. It is possible to use monoclonal antibodies against IL-5. The use of such genetically engineered biological therapy reduces the frequency of exacerbations and the dose of steroids. Thus, we can conclude that in Th2-dependent asthma, eosinophils play the main role of effector cells, and one of the key cytokines is IL-5. However, in the absence of IL-5, eosinophil infiltration and degranulation are determined. It is supported by the vascular cell-1/CC-chemokine/GM-CSF production cascade [24]. Cysteine leukotriene may also directly induce eosinophilic infiltration and airway activation in asthma. Consequently, different mechanisms may be involved in eosinophilic airway inflammation in asthma.

Not only eosinophils but also mast cells or neutrophils play an important role in the pathogenesis of severe asthma. Mast cells largely infiltrate smooth muscle in severe asthma and cause airway remodeling by releasing inflammatory mediators.

Many reasons that underlie the delay or delayed activation of the immune system in COVID-19 are now already known. They include the use with the SARS-CoV-2 virus of strategies to avoid recognition and induce a nonspecific immune response [29,30]. They are associated primarily with a blockade of IFN type I and proinflammatory cytokine production due to suppression of NF-κB transcription factor activity. The presence of certain alleles of class I and/or II MNS molecules in SARS-CoV-2-infected patients may contribute to a decrease in the presentation of viral antigens to the acquired immune system or alleles of ACE2 cell receptors, which ensure high efficiency of infection of host cells [31,32]. However, in most cases of SARS-CoV-2 infection, rapid activation of various cells of the immune system occurs. It is reflected in increased expression of cellular activation markers by monocytes and lymphocytes, appearance of extracellular vesicles of various origins in the peripheral blood and increased levels of key proinflammatory cytokines, and proteins of the acute phase of inflammation [33,34,35,36,37,38]. Elimination of viral infection requires successful cooperation between cellular and humoral factors of the immune system, which is a determining factor for the development of a protective response against the invading pathogen. However, numerous studies have revealed significant impairments in the functioning of various immune cell types, including CD4+ T cells, CD8+ T cells, B cells, NK cells, monocytes, and other circulating, and resident cells in the COVID-19 [39,40,41]. In addition, the difficulty of finding effective therapeutic approaches for COVID-19 is due to the lack of pathognomonic clinical manifestations and different immune reactions in different patients.

### 3.1. Inflammatory Cells

Lymphocytes: Due to a large number of studies of allergic inflammation in animal models, it has turned out that in human asthma there is a predisposition to Th2-associated inflammation, which causes the recruitment of eosinophils to the pathological focus [27]. Furthermore, the production of Th2 cytokines (e.g., interleukin-4 (IL-4), IL-5, and IL-13) may also explain the hyperproduction of IgE and the attraction of eosinophils to the airways [24,42,43].

There may also be a decrease in the number of such subsets of lymphocytes, regulatory T cells, which normally inhibit Th2 cells, and an increase in natural killer (NK) cells, which release large amounts of Th1 and Th2 cytokines [19]. When CD4+ T-cell subpopulations were analyzed in acute COVID-19, there was a decrease in the proportion of Th17.1 and Th1 lymphocytes which are capable of IFN-γ production, as well as a slight decrease in circulating Treg levels [44]. Another study showed a decreased proportion of T helper cells carrying on their surface the key Th17 antigens CD161 and CCR6, while the frequency of T cells expressing CCR4 and GATA3, which are Th2 markers, was higher than in healthy donors, and hyperproduction of Th2 effector cytokines IL-4 and IL-13 was also observed [45]. Moreover, the increased proportion of CCR4-positive Th2 and decreased Th17 and follicular memory Th cells were characterized primarily in patients with a severe course of COVID-19 [46]. Similar results were obtained by Schultheiß et al. who noted an increase in the proportion of Th17 and follicular T cells in the peripheral blood of COVID-19 patients against the background of a slight decrease in Th1, while the values obtained for Th2 and Th17.1 did not differ from the control group [47]. Also, T-lymphocytes may be involved in airway remodeling.

Basophils play an important role in the pathogenesis of various types of bronchial asthma through the production of the widest range of inflammatory mediators [48,49]. Furthermore, these cells are capable of directional migrating to foci of inflammation. They are detected in the airways of patients with asthma according to the results of postmortem biopsies which were found in the lumen of the airways in the intercellular space of the bronchial epithelium and in the submucous membrane [50,51]. Moreover, in response to activation, BALJ-derived basophils were capable of producing more IL-4, responsible for the formation of Th2 cells from “naive” Th0 lymphocytes [52]. Numerous papers indicate a decrease in basophil levels in the peripheral blood of patients in the acute phase of SARS-CoV-2 infection [53,54,55].

Furthermore, basophil levels in patients with an unfavorable outcome were significantly reduced relative to the values of patients with a favorable disease outcome. An inverse correlation among high relative and absolute content of basophils and the risk of developing a severe course of COVID-19 was also revealed, and the recovery level of these cells to a normal frequency can be considered as a prognostic parameter of the transition to the recovery phase [56,57].

Mast cells: The activation of mast cells in the upper airway mucosa leads to the release of bronchoconstrictor mediators such as histamine, cysteinyl-leukotriene, and prostaglandin D2 [21,22]. Allergen activation occurs via the high-affinity IgE receptors and it is the most significant reaction accompanying bronchospasm. Mast cells can also release large amounts of cytokines, altering the airway environment and contributing to inflammation. The effects of allergens are limited. The involvement of mast cells in the pathogenesis of COVID-19 may be related to the release of various inflammatory mediators which are capable of inducing activation of immune system cells and connective tissue in the focus of SARS-CoV-2 virus entry [58]. For example, the levels of mast cell-specific enzymes β-tryptase, chymase, and carboxypeptidase A3 were elevated in patients with acute COVID-19 compared with healthy donors. It was closely associated with increased concentrations of some proinflammatory chemokines (IP-10, CCL2, and CCL4), which allowed us to assess the severity of the course of COVID-19 [59]. Changes in serum levels of mast cell matrix metalloproteinases, MMP-2 and MMP-9, which predicted the risk of hospital-acquired death and severity of disease course, were also observed, suggesting an important pathophysiologic and prognostic role of these molecules and mast cells in acute COVID-19 [60]. More importantly, several independent studies have shown increased numbers of CD117+ mast cells and IL-4-expressing cells in the perivascular space and alveolar septa in biopsy specimens of inflamed lung tissue from patients with acute COVID-19 compared with controls [61,62,63]. 

Thus, this cell type may be involved in the pathogenesis of both diseases, allowing mast cells to be considered as a target for therapy in asthma and acute viral infections, including COVID-19.

Eosinophils: Elevated levels of eosinophils are found in the airways of most asthma patients [31]. These cells contain inflammatory enzymes, produce leukotrienes and express a wide range of proinflammatory cytokines. Elevated eosinophil levels often correlate with severe asthma. In addition, numerous studies show that treatment of asthma with corticosteroids reduces circulating eosinophil levels and the number of eosinophils in the airways in parallel with clinical improvement. However, the role and contribution of eosinophils in asthma is reevaluated, based on studies of anti-IL-5 treatment, which significantly reduces eosinophils but has no effect on asthma control [24]. In the case of acute COVID-19, reduced eosinophil content in peripheral blood was characteristic of most patients and was closely related to the severity of the disease [64,65]. In addition, eosinopenia could be considered as a predictor of the COVID-19 progression, whereas the return of eosinophils to normal values during therapy indicated a favorable course of the disease [66]. It is also interesting to note the fact that low eosinophil counts on hospital admission were associated with high levels of lung tissue lesions on radiographic exacerbation, and these patients had longer hospital stays and a more severe COVID-19 course compared with patients without eosinopenia [67].

Neutrophils: They are elevated in the airways and sputum of patients with severe BA, during exacerbations and when smoking. Regulatory mechanisms of neutrophil activation and changes in lung function continue to be the subject of active study [25]. Increased levels of neutrophils in the circulating blood are one of the most important signs of inflammation in COVID-19 [68,69,70]. Thus, an increase in the level of these cells in the circulation in conjunction with some other routine clinical tests allows us to distinguish patients in critical condition from patients with a severe course of the disease already in the early stages of the development of infection. Moreover, elevated neutrophil levels were observed in patients with severe COVID-19 compared to milder forms of the disease. It could also be used to predict the course of COVID-19 in the hospital setting [71,72]. Furthermore, many researchers noted the appearance of neutrophils with an atypical disturbed phenotype in circulation, including CD16+CD11bhi, CD10LowCD101-CXCR4+/-, as well as CD33++CD16-CD11b-, CD33+CD16-CD11b+, CD33lowCD16+CD11b+, and CD33-CD16+CD11b-low, indicating significant disturbances in the processes of neutrophil maturation in the red bone marrow [73,74,75].

Dendritic cells: The function of dendritic cells is a key interaction with antigen-presenting cells that respond to allergens arriving from the surface of the respiratory tract. Then, the cells migrate to regional lymph nodes [24,25]. In the acute phase of COVID-19, many authors have reported a decrease in circulating blood dendritic cells, affecting both the total pool of these cells and their different subpopulations, including myeloid (CD11c+CD123lo/-) and plasmacytoid (CD11c-CD123+) dendritic cells [76,77,78]. Moreover, all DCs populations possessed a “disturbed” phenotype, which was reflected in reduced expression levels of cell markers which were responsible for antigen presentation and signaling to T lymphocytes, including molecules such as CD80, CD86, and HLA-DR. In addition, the decrease in the functional activity of circulating DCs was long-lasting, as even in already successful COVID-19 patients, these disorders persisted for a long time [78,79].

Macrophages: They are the most numerous cells in the airways that are activated by allergens via low-affinity IgE receptors. It is followed by the release of inflammatory mediators and cytokines that enhance the inflammatory response [27]. Furthermore, in the composition of bronchoalveolar lavage in patients with asthma, an increased level of M2 macrophages was observed. They stimulate fibrosis of surrounding tissues by means of cytokine production [80]. However, in the case of viral infections, it is usually M1 macrophages that play a key role in the body’s defense, capable of producing a huge amount of proinflammatory factors into the inflamed tissue [81]. For this reason, in the case of SARS-CoV-2 infection, M1 macrophages were dominant in the lung tissue of patients [82]. Further, in the peripheral blood of patients with acute COVID-19, cells of mixed M1/M2 phenotype within intermediate and non-classical monocyte populations were detected. In the authors’ opinion, they indirectly indicated prolongation of inflammation and development of fibrosis as a repair mechanism. However, they damaged the lung parenchyma and potentially increased the risk of worsening clinical outcomes [83].

Smooth muscle cells: Airway smooth muscle is not only targeted in the asthmatic response (by contracting and causing airway obstruction), but also contributes to its development. The activation and release of related proinflammatory mediators occur [26]. Against the background of airway inflammation and the production of growth factors, airway smooth muscle cells undergo proliferation, activation, contraction, and hypertrophy. These processes may influence airway dysfunction in asthma. It should be noted that airway smooth muscle contraction plays a critical role in effective ventilation and it is influenced by several cytokines, most notably IL-13 secreted by Th2 cells and IL-17 produced by Th17 cells [84]. This is particularly important for inflammatory processes affecting lung tissue in the context of the “cytokine” storm in the COVID-19. Moreover, analysis of biopsy specimens of affected lungs from patients with the COVID-19 revealed a dramatic decrease in smooth muscle cells in foci of inflammation [85]. This process could be associated with impaired proliferation and differentiation of these cells. In conditions of “cytokine” storm, IL-6 can be produced by vascular smooth muscle cells, which may also contribute to the development of local inflammatory response in the COVID-19 [86].

Resident myeloid dendritic cells: Airway smooth muscle is not only the target of the asthmatic response, which is accompanied by its contraction and airway obstruction, but it also contributes to its development [26]. As a result of airway inflammation, growth factor production and airway smooth muscle cells can undergo proliferation, activation, contraction, and hypertrophy. These events may influence airway dysfunction in asthma.

Epithelial cells: These cells are lining the airways. Activation and production of inflammatory mediators, attraction, and increase in the level of inflammatory cells, including against the background of infection with respiratory viruses, leads to the production of inflammatory mediators by epithelial cells with damage to the epithelium [20]. The process of recovery from epithelial damage can be impaired in asthma, which is accompanied by obstruction. In the COVID-19 background, lung epithelial cells are one of the main targets for the influence of SARS-CoV-2 virus because they have ACE2 receptors on their surface. These and other receptors promote virus entry into cells, stimulating not only oxidative stress and inflammation, but also promoting the expression of soluble profibrotic factors. These factors are responsible for the activation and recruitment of fibroblasts, their transformation into myofibroblasts, and the unregulated production of intercellular matrix proteins [87]. At the same time, IL-13 is able to suppress ACE2 expression on the surface of epithelial cells. It reduces the efficiency of their virus infection and indirectly indicates an important role of Th2 cytokines in defense against respiratory infections, including SARS-CoV-2 [88]. Moreover, it is in patients with asthma that reduced ACE2 expression on the surface of lung epithelial cells is observed [89,90]. As shown by Morrison et al, in vitro stimulation of bronchial airway epithelial cells with IL-13 significantly reduces virus release and epithelial cell damage, which may be associated with reduced SARS-CoV-2 virus entry and replication [91]. At the same time, Th2 cytokines can reduce the efficiency of type I and III IFN production by epithelial cells, thereby reducing the effectiveness of the antiviral response as a whole [92].

### 3.2. Inflammatory Mediators

Chemokines: Eotaxin is relatively selective for eosinophils. At the same time, thymic activation-regulated chemokine (TARC) and chemokines (MDC) derive from macrophages and recruit Th2 cells.

Cytokines: They direct and regulate the inflammatory response in asthma and determine its severity. Cytokines, produced by Th2, activate IL-5 which is essential for the differentiation and survival of eosinophils. IL-4 is important for Th2 cell differentiation and is also activated. IL-13 is also important for IgE formation [28]. Key cytokines include IL-1β and tumor necrosis factor-α (TNF-α), which enhance the inflammatory response. They activate granulocyte–macrophage colony-stimulating factor (GM-CSF) and it prolongs the survival of eosinophils in the airways [20,24].

Cysteinyl-leukotrienes: These mediators are potent bronchoconstrictors originating mainly from mast cells. They are the only mediators whose inhibition is specifically associated with improved lung function and relief of asthma symptoms [28]. 

Nitric oxide: It is formed primarily by the action of inducible NO synthase in airway epithelial cells; it is a potent vasodilator [33]. Measurement of fractional content of NO in exhaled air (FeNO) is useful in monitoring the response to asthma treatment, due to the presumed link between FeNO and the presence of inflammation in asthma. This indicator is now being actively adopted in the treatment of asthma in children [93].

Immunoglobulin E: It is an antibody that is responsible for the development of allergic reactions and plays an important role in the pathogenesis of allergic diseases and in the development and maintenance of inflammation. IgE attaches to the cell surface through a specific receptor with high affinity. The mast cell has a large number of IgE receptors which are activated by interaction with antigen and release a wide range of mediators. These processes provoke acute bronchospasm and release proinflammatory cytokines that support the underlying airway inflammation [17]. Other cells, basophils, dendritic cells, and lymphocytes also have high-affinity IgE receptors.

The development of anti-IgE monoclonal antibodies has shown that their administration causes a reduction in IgE levels, which is effective in the treatment of asthma [94]. The findings further support the importance of IgE in asthma. Thus, our understanding of asthma pathogenesis and underlying mechanisms now includes the concept that gene–environment interactions are a critical factor in the development of airway inflammation and possible changes in lung physiology. These changes and factors determine the immune response and characterize the severity of the course of asthma.

## 4. Predisposing Factors

Currently, the provoking factors for the development and activation of BA are presented in various studies [95,96].

### 4.1. Genetic Predisposition

The role of genetic predisposition in bronchial asthma is well known [27]. Currently, many genes have been identified that are involved or associated with the development of asthma and they determine the characteristics of its course [97]. The complexity of their involvement in the clinical course of asthma is determined by their association with certain phenotypic characteristics. However, these processes are not necessarily associated with the pathophysiological process of the disease or the clinical picture itself [98].

### 4.2. Gender

Asthma is more commonly diagnosed in boys at an early age. However, during puberty, the sex ratio changes and asthma appears predominantly in women [97,98]. The relationship between sex hormones and the development of asthma has not yet been established. At the same time, they can contribute to the onset and maintenance of the disease.

### 4.3. Obesity

Increasing rates of obesity have been accompanied by an increase in the prevalence of asthma, but the relationship is unclear [99,100]. Obesity may be a risk factor for asthma because of the production of unique inflammatory mediators that lead to airway dysfunction.

### 4.4. Allergens

Allergens determine the development of bronchial asthma. Exposure to house dust mites is an important factor in the development of asthma in children [101,102]. However, recent studies have shown that in some cases, early exposure to dogs and cats may actually protect against the development of asthma. The factor that is responsible for these different results has not been established. Studies evaluating exposure to house dust mites and cockroaches have shown that the sensitization and subsequent development of asthma correlate with allergen exposure. They contribute to the persistence of airway inflammation and increase the likelihood of exacerbation [103].

### 4.5. Air Pollution

One of the important areas of research is the study of the influence of environmental pollutants on the development and severity of bronchial asthma [104]. The observed increase in the prevalence of BA in countries with a high level of development and urbanization clearly correlates with the growth of industrial development in these countries [105,106]. Air pollution in the development of asthma remains controversial and may be associated with allergic sensitization [105]. Epidemiological studies have shown that outdoor sports in communities with high ozone concentrations are associated with a higher risk of asthma development among school-aged children [107]. The relationship between elevated pollution levels and increased asthma exacerbations and emergency department visits is well established.

### 4.6. Professional Factors

It was found that chronic cough with sputum secretion occurred more often in people who were in contact with harmful factors, and it was in this population that cases of first-onset bronchial asthma were registered. At the same time, it was found that even when contact with harmful occupational factors was reduced, nonspecific bronchial hyperreactivity did not disappear over time in people with occupational asthma [108]. The severity of occupational asthma is determined mainly by the duration of the disease and the severity of symptoms. These asthma manifestations are independent of age, sex, occupational hazards, and smoking [109,110].

### 4.7. Smoke

Tobacco smoke, air pollution, exercise, and diet are also associated with an increased risk of asthma. However, the association of asthma with these factors is not as well established as with allergens and respiratory infections [111].

Intrauterine exposure to tobacco smoke increases the likelihood of obstruction in a child. The subsequent development of asthma is not well established. In adults with asthma, cigarette smoking is associated with increased asthma severity and decreased sensitivity to inhaled corticosteroids (ICS) [100]. E-cigarette use is associated with an increased risk of respiratory symptoms and asthma exacerbations [112,113].

### 4.8. Nutrition

Studies in many countries, aimed at determining the influence of diet on the course of the disease, have shown that individuals consuming foods of plant origin, juices rich in vitamins, fiber, and antioxidants have a slight tendency to a more favorable course of asthma [114]. Consumption of animal products rich in fats, proteins, and refined easily digestible carbohydrates is associated with a severe course of the disease and frequent exacerbations [115].

### 4.9. Viral Infections

The significance of exposure to respiratory viruses in the development of bronchial asthma is greatest in early childhood. In early childhood, respiratory syncytial virus (RSV) and parainfluenza virus in particular cause of bronchiolitis, most often in childhood [42,109].

At the same time, evidence suggests that some early respiratory infections, including measles or recurrent viral infections (other than lower respiratory tract infections) [116], may protect against the development of asthma. The current “Hygiene Hypothesis” of asthma states that exposure to infections at an early age affects the development of the child’s immune system in a “non-allergic” manner, resulting in a reduced risk of asthma and other allergic diseases. Although the hygiene hypothesis continues to be investigated, this link may explain the observed association between larger family size, later birth order, kindergarten attendance, and reduced risk of asthma.

The impact of acute respiratory infections on the development of asthma may depend on the interaction with atopy. The atopic condition may influence the response of the lower respiratory tract to viral infections, and viral infections may then influence the development of allergic sensitization. Respiratory interactions that may occur when people are simultaneously exposed to allergens and viruses are of interest but it is currently not studied. Of particular interest is the impact of SARS-CoV-2 and varying severity of COVID-19 on the development of asthma.

### 4.10. SARS-CoV-2 Infection and Disease Severity Bronchial Asthma

According to the results of these studies, a protective role of some factors in the development and severe course of COVID-19 in BA patients has been shown [117,118]. Other studies have shown that severe asthma was associated with a higher risk of death from COVID-19. Patients with asthma required intensive care in adults, admitted to hospital with COVID-19 [10]. Long-term outcome studies are limited, although in a population-based symptom study, asthma was the only pre-existing condition associated with an increased risk of long-term COVID-19 disease. Improved understanding of how COVID-19 affects people with asthma, both acutely and in the long term, is vital to ensure the adequacy of the current pandemic response.

A systematic review and meta-analysis data introduced 131 studies where it was suggested that the development of asthma was not associated with COVID-19 severity. In addition, patients with BA tended to have a lower risk of death compared with patients without asthma, and there was no increase in the need for intubation and mechanical ventilation [11].

Alternative data were obtained in another study with analysis of 150 publications, where an increased incidence of COVID-19 detection and a high risk of hospitalization, severity, or mortality in patients with BA were proven [119]. Among patients treated in the ICU with SARS-CoV-2 pneumonia who required artificial ventilation, there was a low prevalence of BA (1.8%) [120].

At the same time, an increased risk of COVID-19-related death was observed among patients with asthma who received high-dose glucocorticoid therapy. At the same time, according to the authors, the observed increased risk of death associated with COVID-19 could be explained by the influence of disease severity and exacerbations, as well as other significant clinical factors that may have influenced the results of the study [121].

A multicenter prospective cohort study found that patients with asthma were more likely to receive intensive therapy than patients without background respiratory disease. In patients aged 16 years and older, severe BA was correlated with a high rate of death compared with non-severe asthma [10].

According to the data of J. Ren et al. (2022) in a national prospective study, asthma was associated with a lower risk of COVID-19 infection; this association was not statistically significant and was observed in patients under 65 years of age. In contrast, in elderly patients, BA did not affect the risk of COVID-19 infection [121]. However, a diagnosis of BA was associated with a greater likelihood of hospitalization for COVID-19 at any age [122].

According to analysis data, people with asthma are at lower risk of COVID-19 infection compared with people without asthma, but have the same risk of hospitalizations, ICU admissions, ventilator use, and mortality for positive reverse transcription polymerase chain reaction (RT-PCR) [123].

Some studies have suggested that there is a large difference between the number of COVID-19 infections in patients with asthma in different geographic regions [123]. Several factors explain these differences: different reactivity to COVID-19 infection and differences in asthma prevalence among different races or ethnic groups [124]. Clearly, asthma phenotypes are important for assessing the risk of SARS-CoV-2 infection and disease severity [125].

Th2 inflammation suppresses the production of proinflammatory cytokines such as IL-1β, TNF-α, and IL-6, which are important for defense against viral infections, but also play a role in the late phase of hyperinflammation, which is the typical phase of COVID-19 leading to severe disease [126]. Several studies have shown that patients with non-allergic asthma have a higher risk of testing positive for SARS-CoV-2 and a higher risk of COVID-19 severity, whereas patients with allergic asthma do not have a significantly increased risk of COVID-19 morbidity and severity [127,128]. Meanwhile, high Th2 inflammation may reduce the risk of SARS-CoV-2 infection and determine disease severity, in contrast to high-risk individuals in patients with asthma and low Th2 levels [129]. M1-like macrophages make an important contribution to antiviral immunity in patients with asthma. Their number is reduced in patients with allergic asthma during exacerbations caused by viral infection, such as that caused by rhinovirus [27,129]. This mechanism may be one of the explanations why clinical and pathologic outcomes, particularly rhinovirus infection, are more severe in allergic patients than in healthy individuals [130].

## 5. Bronchial Asthma and COVID-19 Treatment

It is known that glucocorticosteroids (GS) exert anti-inflammatory effects in the lungs, reduce ACE-2 and TMPRSS2 expression in bronchial epithelial cells, and may reduce SARS-CoV-2 replication in epithelial cells in vitro. This has contributed to the hypothesis of a protective role of IGS in SARS-CoV-2 infection [125].

In an open-label randomized and controlled trial of steroids in the treatment of COVID-19 (STOIC) in parallel groups (phase 2), administrating inhaled budesonide to adults with early-stage COVID-19 reduced the likelihood of requiring emergency care, emergency department consultation, or hospitalization, with a faster resolution of symptoms [131]. The findings warranted further studies. In a randomized controlled open-label, adaptive-platform open trial (PRINCIPLE), inhaled budesonide reduced symptoms severity and shortened recovery time with a trend toward fewer hospitalizations or mortality (although the results did not meet the threshold for superiority) in high-risk COVID-19 patients [132]. Another study found that glycopyrronium, formoterol, and the combination of glycopyrronium, formoterol, and budesonide inhibited coronavirus 229E (HCoV-229E) replication. It exacerbates the course of bronchial asthma, in part by inhibiting receptor expression and/or endosome function, and modulated infection-induced inflammation in the airways [133].

However, opinions regarding the use of oral corticosteroids are conflicting. On the one hand, the treatment of severe asthma may sometimes require long-term use of oral corticosteroids, and abrupt discontinuation of oral corticosteroids may be dangerous [3]. Oral steroids should continue to be used to treat severe asthma exacerbations [120]. On the other hand, chronic or relapsing use of oral corticosteroids prior to SARS-CoV-2 infection is a major risk factor for poor COVID-19 treatment outcomes and worse survival [3].

In the treatment of severe uncontrolled asthma, the efficacy and safety of therapeutic approaches are determined by targeted biologic therapy. According to GINA recommendations, patients who are receiving biologic therapy should continue treatment with these drugs in the same volume [3,133].

The pioneer among all monoclonal antibodies, used in asthma patients, is omalizumab. Patients with severe BA, especially children, should be referred for expert observation to assess the severity of the disease and the possibility of immunotherapy. Therapy with omalizumab (humanized anti-IgE monoclonal antibody), developed on the basis of human IgG1 with a kappa-light chain, IgE binding provided by mouse antibody sites, is recommended for adults, adolescents, and children over 6 years of age with severe asthma of allergic genesis in the absence of efficacy of controlled treatment used at the fourth step [133].

In an observational study, Lommatzsch M. et al. (2020) found that omalizumab not only achieved symptom control but also reduced the risk of viral-induced exacerbations of the disease, probably due to enhanced antiviral immunity. According to the authors, allergic asthma or omalizumab, or both, may have had a protective effect in the patient during the course of COVID-19 [134]. However, a systematic review of data from 155 publications provided evidence of reduced incidence and severity of upper respiratory tract infections and asthma exacerbations with omalizumab. Nevertheless, there are insufficient data on the possible positive effect of anti-IgE therapy on COVID-19 and the need for new studies to prove this effect [135].

Patients with the T2 endotype of asthma may have a lower risk of COVID-19 infection due to higher eosinophil counts and reduced ACE2 expression. Treatment of patients with the T2 endotype with omalizumab may be an additional protection against COVID-19 because the role of IgE in reducing the antiviral response and the beneficial effects of anti-IgE therapy on the immune system are well understood. The use of omalizumab as a potential treatment for COVID-19 needs to be investigated [136,137].

## 6. Conclusions

COVID-19 has become a global threat to the health of the planet’s population on the one hand. It has intensified the study of new pathological conditions and immunological changes associated with the development of this infection on the other hand. Extensive organ damage is known to occur in COVID-19, which is explained by the expression of ACE2 (angiotensin-converting enzyme 2). SARS-CoV-2 can enter cells expressing ACE2, but not cells lacking ACE2 or cells expressing other coronavirus receptors such as aminopeptidase N and dipeptidyl peptidase 4 (DPP4), suggesting that ACE2 is a cellular receptor for SARS-CoV-2 [19].

It is known that there is a relationship between various trigger factors that provoke the development of bronchial asthma. There is currently evidence that the SARS-CoV-2 virus may be one of the triggers of asthma. Inflammatory mediators, recruitment and activation of inflammatory cells, and infection with respiratory viruses can lead to the production of more inflammatory mediators or damage the epithelium itself [20]. The process of recovery from epithelial damage in asthma may be abnormal, contributing to the obstructive lesions that occur in asthma. In the case of COVID-19, lung epithelial cells are one of the main targets for SARS-CoV-2, as they display ACE2 receptors on their surface, through which the virus enters the cells, stimulating not only oxidative stress and inflammation, but also promoting the expression of soluble profibrotic factors which are responsible for the activation and recruitment of fibroblasts, their transformation into myofibroblasts, and the unregulated production of intercellular matrix proteins.

COVID-19 has affected the course of asthma. However, until now there is no clear idea of whether asthma progresses during or after COVID-19 infection. According to the results of some studies, a significant difference was identified between the development of bronchial asthma in people after COVID-19 [123]. Several factors explain this difference, including different vulnerability to COVID-19 infection and differences in asthma prevalence among different races or ethnic groups [124]. Clearly, asthma phenotypes are important for assessing the risk of SARS-CoV-2 infection and disease severity [125]. Mild to moderate asthma is likely not associated with COVID-19 severity. One of the protective factors that prevents the development of severe bronchial asthma under the influence of SARS-CoV-2 infection is the use of glucocorticoid therapy. Targeted asthma therapy using omalizumab may help treat severe asthma due to COVID-19 [137]. Evidence has now been obtained for the efficacy of omalizumab on reducing the level of IgE biological to reduce the number of asthma exacerbations caused by respiratory viruses. The drug reduces the number of attacks, their duration, and severity in patients with asthma. The accumulated experience with omalizumab in patients with severe asthma during COVID-19 who received omalizumab during the pandemic has strongly suggested that continued treatment with omalizumab is safe and may help prevent the severe course of COVID-19. Accordingly, in guidelines issued by the Global Asthma Initiative, it was recommended that all patients with asthma continue to take their prescribed asthma medications, including biologic therapy, during the COVID-19 pandemic. The impact of biologic treatment in patients with asthma and COVID-19 will be better understood as more knowledge is gained. Data analysis should be continued, especially taking into account the results of the course of bronchial asthma after COVID-19 of varying severity.

## Data Availability

Not applicable.
